# Haplotype-specific PCR for *NAT2* diplotyping

**DOI:** 10.1038/s41439-020-0101-7

**Published:** 2020-05-11

**Authors:** Nuanjun Wichukchinda, Jirapa Pakdee, Punna Kunhapan, Wimala Imunchot, Licht Toyo-oka, Katsushi Tokunaga, Surakameth Mahasirimongkol

**Affiliations:** 1 Department of Medical Sciences, Ministry of Public Health, Nonthaburi, Thailand; 2 Department of Human Genetics, Graduates School of Medicine, The University of Tokyo, Tokyo, Japan; 3 Present Address: National Bioscience Database Center, Japan Science and Technology Agency, Tokyo, Japan; 4 Present Address: Genome Medical Science Project, National Center for Global Health and Medicine, Tokyo, Japan

**Keywords:** Predictive markers, Risk factors

## Abstract

N-acetyltransferase 2 (NAT2) is an enzyme that acetylates many kinds of drugs, including the antituberculosis drug isoniazid. The *NAT2* gene is highly diverse across populations. An individual can be classified as having a slow acetylator (SA), an intermediate acetylator (IA), or a rapid acetylator (RA) phenotype based on its two haplotypes (diplotype) of *NAT2*. SA individuals are at a higher risk for isoniazid-induced hepatitis, while the RA phenotype contributes to failure in tuberculosis treatment. Being able to predict individual NAT2 phenotypes is important for dose adjustment of isoniazid. *NAT*2 haplotypes are commonly determined via an indirect method of statistical haplotype inference from SNP genotyping. Here, we report a direct *NAT2* haplotyping method using haplotype-specific PCR (HS-PCR) for the 6 most commonly found *NAT*2 haplotypes: *NAT*2*4*, NAT*2*5*B, NAT*2*6*A, NAT*2*7*B, NAT*2*12*A*, and *NAT*2*13*A*. Validation of this HS-PCR method via comparison with a sequencing method in 650 Thai DNA samples (107 RA, 279 IA, and 264 SA samples) showed a concordance rate for diplotype calls of 99.23% (645/650 samples). The discordant results in 5 samples were due to 3 rare *NAT2* haplotypes: NAT*5*C* (*n* = 3), *NAT*2*7*C* (*n* = 1), and *NAT*2*11*A* (*n* = 1). This novel HS-PCR method allows direct *NAT*2 diplotyping, enabling the implementation of NAT2 acetylator phenotypes in clinical pharmacogenetic testing.

## Precision medicine: Improved genotyping to predict drug response

Physicians will be able to quickly and affordably personalize the dosage of the anti-tuberculosis drug isoniazid thanks to a new genotyping technique. Variation in the *NAT2* gene affects how people metabolize many drugs, including isoniazid. An international team led by Nuanjun Wichukchinda of Thailand’s Deapartment of Medical Sciences, Ministry of Public Health, have developed a direct haplotyping polymerase chain reaction (PCR) approach to determine which version of *NAT2* an individual carries. The technique uses DNA sequences specific to the most common variants in the Thai population. The results of the PCR approach had a 99% match with results from sequencing, which is slower, more expensive, and requires more complicated machinery. With this approach, patients can be routinely genotyped to identify their version of *NAT2*, and the dosage of isoniazid and other drugs metabolized by *NAT2* adjusted accordingly.

## Introduction

N-acetyltransferase 2 (NAT2) is a liver enzyme necessary for the detoxification and metabolism of foreign chemicals and various drugs, such as caffeine, isoniazid, sulfamidine, hydrazine, dapsone, procaine amide, sulfapyrimidine, nitrazepam amide, sulfapyridine, and nitrazepam^1–4^.

The *NAT*2 gene is highly diverse, with 20 clusters (108 haplotypes) reported in the global population^1,5^. *NAT*2*4 is considered the reference and encodes a high-enzymatic activity product. The other *NAT*2 haplotypes are assigned according to their SNP variances in exon 2^5^. Haplotype inference is usually derived from the seven most common SNPs, namely, rs1801279 (191G > A), rs1041983 (282C > T), rs1801280 (341T > C), rs1799929 (481C > T), rs1799930 (590G > A), rs1208 (803A > G), and rs1799931 (857G > A), which are the signature SNPs for the following 7 clusters: NAT2*14, NAT2*13, NAT2*5, NAT2*11, NAT2*6, NAT2*12, and NAT2*7, respectively. *NAT2* haplotypes containing the nonsynonymous SNPs 191G > A (R64Q), 341T > C (I114T), 590 G > A (R197Q), and 857 G > A (K268R) encode low-activity or slow acetylation enzymes. Common slow acetylation haplotypes include NAT2*5B, NAT2*6 A, NAT2*7B, and *NAT*2*14A/*14*B*, whereas *NAT*2*12*A* and *NAT*2*13*A* encode high-activity enzymes similar to those from reference *NAT*2*4^5^. According to the codominance of two *NAT2* haplotypes (diplotype), individual phenotypes are classified as rapid acetylator (RA), intermediate acetylator (IA), or slow acetylator (SA). The distribution of NAT2 haplotypes and the proportions of SA, IA, and RA vary among ethnic groups^5^.

Tuberculosis (TB) is one of the most serious health problems, with more than 2 billion infected people (approximately one-third of the world’s population) and an estimated 10 million new cases occurring in 2017^6^. In Thailand, more than 100,000 patients have been diagnosed with TB, and 12,000 patients die every year^7^; hence, precise diagnosis and treatment are necessary to control TB. Anti-TB drugs are usually combined drugs that are administered for a 2-month period. The first-line anti-TB drug is composed of isoniazid, rifampicin, pyrazinamide, ethambutol, and streptomycin. The most common adverse reaction to anti-TB drugs is skin rash (15.4%); hepatitis is the second most common (9.2%) adverse effect, but it is more life-threatening than skin rash and causes treatment failure^8,9^.

Patients with the SA phenotype are prone to adverse effects from drugs metabolized by NAT2. A meta-analysis that included 14 studies^10^ showed that the risk of anti-TB drug-induced liver injury (AT-DILI) was higher for the SA type than for other acetylator types (OR = 4.695, 95% CI: 3.291–6.705, *p* < 0.001). This finding was confirmed in a study of the Thai population^11^ that also explored AT-DILI (OR = 8.80, 95% CI: 4.01–19.31, *p* = 1.53 × 10^−8^). Therefore, being able to determine a patient’s NAT2 acetylator type would help physicians adjust the dosage of isoniazid.

*NAT*2 haplotypes are conventionally determined by inference from seven common SNPs located in exon 2 of the *NAT2* gene. SNP genotyping methods, such as sequencing^12^, real-time polymerase chain reaction (PCR)^13^, PCR-restriction fragment length polymorphism^14,15^, allele-specific sequencing^16^, and allele-specific primer extension^17^, have complicated steps, are laborious, time-consuming and costly, and/or require sophisticated machines. These resource requirements limit the routine use of such methods in clinical applications. Furthermore, statistical inference may be error-prone and difficult for nonstatisticians to conduct. Therefore, we aimed to develop a simple and low-cost method for *NAT2* diplotyping that directly provides 2 haplotypes without an inference step and can therefore be used in routine service.

## Materials and methods

Frozen EDTA blood samples (*n* = 650) from stocks available from the Third Thailand National Health Examination Survey Program were randomly selected to represent the Thai populations of 13 health areas. The survey was approved by the Ethical Review Committee for Research in Human Subjects, Ministry of Public Health^18^, and all participants provided written informed consent. We obtained permission to use these frozen blood samples with information on sex and residential area. DNA samples were extracted using a commercial kit (QIAamp DNA blood mini kit, QIAGEN GmbH, Germany) and quantitated using a spectrophotometer (Nanodrop-100, Wilmington, DE 19810, USA).

The haplotype-specific PCR-based method (HS-PCR) for *NAT2* diplotyping presented here uses 6 reaction tubes, with each tube containing a specific primer pair for one of the haplotypes most commonly found in Thai populations (NAT2*4, NAT2*5*B, NAT*2*6*A, NAT*2*7*B, NAT*2*12*A*, and *NAT*2*13*A*). These oligonucleotide primers were designed using *NAT*2*4 (NG_012246.1 *Homo sapiens* N-acetyltransferase 2 (*NAT*2), RefSeqGene on chromosome 8) as a reference sequence. Specific amplification of only one *NAT*2 haplotype was performed by using a combination of forward and reverse primers that contained a haplotype signature SNP as the last base at the 3′ end of the oligonucleotide primer, and its paired primer also contained a specific base at the 3′ end. The six variant bases of these six common *NAT2* haplotypes and the last 3′ end base of each primer are provided in Table 1. Since primers with only a single 3′ mismatched base may give false-positive results, the amplification refractory mutation system^19,20^ was used to introduce an additional mismatched base at the −2 position from the 3′ end of the primer to increase the specificity of HS-PCR. A primer pair (TIMP1-Fw/TIMP1-Rv) for amplification of *TIMP*1, a gene located on chromosome X, was used as an internal control in every reaction tube. The primer sequences used to amplify specific *NAT2* haplotypes and *TIMP*1 and the amplified product sizes are shown in Table 2. The final PCR (12 μl) was composed of 1X ready mix reagent (KAPA2G Fast Multiplex Mix, KAPA Biosystems, Boston, Massachusetts, USA), the *NAT*2-specific primer pair (0.3 μM), the *TIMP*1 primer pair (0.1 μM), and 20–50 ng of a DNA sample. After denaturation at 95 °C for 5 min, 35 cycles of amplification (95 °C for 20 s, 65 °C for 20 s, and 72 °C for 30 s) were performed. The primer sequences used to amplify specific *NAT2* haplotypes and *TIMP1* and the amplified product sizes are shown in Table 2. The six *NAT2* haplotypes were directly determined by evaluating the specific sizes of the PCR products in 1.5% agarose gels stained with ethidium bromide.

**Table 1:** The 7 most common SNPs in *NAT2* exon 2.

SNP position							
NAT2 haplotype	191	282	341	481	590	803	857
	rs1801279	rs1041893	rs1801280	rs199929	Rs1799930	rs1208	rs1799931
NAT2*4	G	C^Fw^	T	C	G	A^Rv^	G
NAT2*5B	G	C	C^aFw^	T	G	G	G^Rv^
NAT2*6 A	G	T	T^Fw^	C	A^aRv^	A	G
NAT2*7B	G	T	T	C^Fw^	G	A	A^aRv^
NAT2*12 A	G	C	T	C^Fw^	G	G^aRv^	G
NAT2*13 A	G	T^aFw^	T	C	G^Rv^	A	G^Rv^

^a^Indicates the signature SNP for each *NAT2* cluster. ^Fw^ indicates the last base at the 3′ end of the forward primer, and ^Rv^ indicates the last base at the 3′ end of the reverse primer. SNP positions were identified by designating “A” of the “ATG” start codon as the first position.

**Table 2:** Primers and sequences used in *NAT2* haplotype-specific PCR and direct sequencing.

*NAT*2 haplotype	Primer name	bp	5′–3′ sequence	PCR product (bp)
	4F-282C	36	GGT TTT CAG ACC ACA ATG TTA GGA GGG TAT TTT GAC	588
*NAT*2*4	4R-803A	38	CAC GAG ATT TCT CCC CAA GGA AAT CTT AAA TAT ATG TT	
	5F-341C	30	CAT GGT TCA CCT TCT CCT GCA GGT GAC AAC	582
*NAT*2*5*B*	5R-857G	35	TTT ATT TTG TTC CTT ATT CTA AAT AGT AAG GGC TC	
	6F-341T	22	ACC TTC TCC TGC AGG TGA CAA T	300
*NAT*2*6*A*	6R-590A	28	TTC ATA GAC TCA AAA TCT TCA ATT GGT T	
	7F-481C	26	GAC AGA AGA GAG AGG AAT CTG GTC CC	431
*NAT*2*7*B*	7R-857A	35	TTT ATT TTG TTC CTT ATT CTA AAT AGT AAG GGC TT	
	12F-481C	26	GAC AGA AGA GAG AGG AAT CTG GTC CC	387
*NAT*2*12*A*	12R-803G	38	CAC GAG ATT TCT CCC CAA GGA AAT CTT AAA TAT ATG TC	
	13F-282T	29	AGA CCA CAA TGT TAG GAG GGT ATT TTG AT	
*NAT*2*13*A*	13R1-590G	28	TTC ATA GAC TCA AAA TCT TCA ATT GGT C	366
	13R2-857G	36	GTT TAT TTT GTT CCT TAT TCT AAA TAG TAA GGG CTC	641
*TIMP*1	TIMP1-F	29	AGT TTC TCA TTG CTG GTG AGG CAC CGT CC	817
	TIMP1-R	29	AGC CAT CAG GGA ACA GGC TTG GAC TAG CC	
*NAT*2-*Exon*2	PCR-Fw	23	CAT GTA AAA GGG ATT CAT GCA GT	1315
	PCR-Rv	23	TAG CAT GAA TCA CTC TGA TTC CC	
	Seq-Fw	25	CAG GTC AAT CAA CTT CTG TAC TGG G	
	Seq-Rv	29	GCA CAT AAG TTG ATA ATT AGT GAG TTG GG	

For *NAT*2 direct sequencing, the PCR-Fw/PCR-Rv primer pair (shown in Table 2) was used to amplify the whole exon 2 of *NAT*2. A 10-μl PCR containing 1X KAPA2G Fast Multiplex Mix, 0.2 μM each primer, and 10–20 ng of DNA was performed under the following conditions: 95 °C for 5 min and 35 cycles at 95 °C for 20 s, 65 °C for 15 s, and 72 °C for 60 s. The amplified products were treated with exonuclease I and alkaline phosphatase (Illustra ExoProStar, GE Healthcare UK Ltd., Buckinghamshire, England) at 37 °C for 30 min and then 95 °C for 15 min to remove unincorporated primers and dNTPs. The sequencing reactions were performed by using 1 ml of the treated PCR products, sequencing primers (shown in Table 2), and a 1X BigDye Terminator v3.1 Cycle Sequencing Kit (Applied Biosystems, Foster City, CA, USA). After purification (D-Pure^TM^ Dye Terminator Removal Kit, NimaGen BV, Nimagen, NL), the sequences were read by a 3500XL genetic analyzer (Applied Biosystems). The variants in exon 2 of *NAT2* were called by Variant Reporter^TM^ Software v2 (Applied Biosystems). The *NAT2* haplotype was inferred based on the most common 7 SNP positions using PHASE software^21,22^.

### Acetylator phenotype interpretation

Since each individual carried two haplotypes (diplotype) in a codominant fashion, analysis of the relationships between *NAT2* diplotypes and acetylator phenotypes was performed as follows. Homozygous or heterozygous genotypes among fast *NAT2* haplotypes (NAT2*4/*4, NAT2*4/*12A, NAT2*4/*13A, NAT2*12A/*12A, NAT2*12A/*13A, and NAT2*13A/*13A) were interpreted as RAs. Homozygous or heterozygous genotypes among slow *NAT2* haplotypes (NAT2*5B/*5B, NAT2*5B/*6A, NAT2*5B/*7B, NAT2*6A/*6A, NAT2*6A/*7B, and NAT2*7B/*7B) were interpreted as SAs. Heterozygous genotypes between fast and slow *NAT2* haplotypes (NAT2*4/*5B, NAT2*4/*6A, NAT2*4/*7B, NAT2*5B/*12A, NAT2*5B/*13A, NAT2*6A/*12A, NAT2*6A/*13A, NAT2*7B/*12A, and NAT2*7B4/*13A) were interpreted as IAs.

## Results

### Direct *NAT*2 diplotyping by HS-PCR

**Fig. 1:**
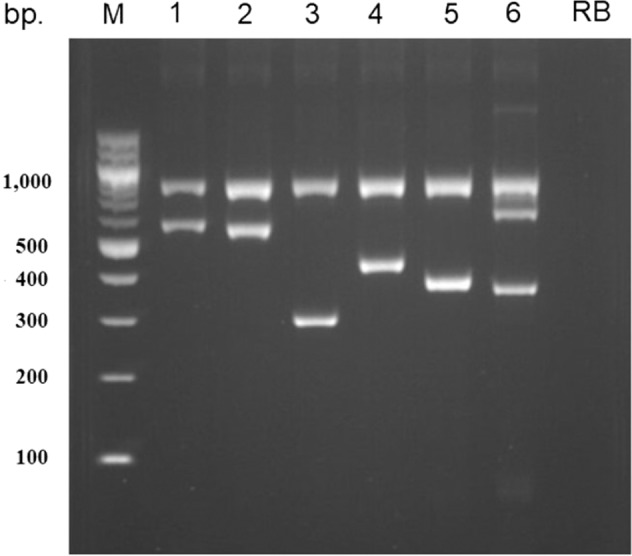
Gel photograph of haplotype-specific PCR. All six reaction tubes contain an internal control of the *TIMP1* PCR product (817 bp). 1 = *NAT*2*4 (588 bp), 2 = *NAT*2*5*B* (582 bp), 3 = *NAT*2*6*A* (300 bp), 4 = *NAT*2*7*B* (431 bp), 5 = *NAT*2*12*A* (387 bp), and 1 = *NAT*2*13*A* (366 and 641 bp). M = DNA ladder and RB = reagent blank.

A novel method for genotyping *NAT2* diplotypes was developed using *NAT2* HS-PCR. The detection range of this method is 5–200 ng of DNA (data not shown). For each *NAT2* haplotype, the specific amplified product size can be clearly and directly observed under UV light after agarose gel electrophoresis and Et–Br staining (Fig. 1). Each NAT2-haplotype reaction tube produces a specific band when a sample is positive for that particular haplotype, except for the NAT2*13 reaction tube, which produces 2 bands of 366 bp and 641 bp from one forward primer and 2 reverse primers. The concordance rate for diplotyping between the novel HS-PCR method and the indirect sequencing method among 650 DNA samples was 99.23% (645/650). The discordant results observed for five samples were due to rare *NAT2* haplotypes comprising *NAT*2*5*C* (three samples), *NAT*2*7*C* (1 sample), and NAT2*11A (1 sample), which were designated *NAT*2*5*B*, *NAT*2*7*B*, and *NAT*2*4 by HS-PCR, respectively. However, in all 650 samples, the interpretation of acetylator phenotypes from *NAT2* diplotypes by the HS-PCR method was 100% concordant with that from the direct sequencing method.

### Frequency of *NAT2* haplotypes and acetylator phenotypes in the Thai population

The *NAT2* haplotypes found by Sanger sequencing and haplotype inference in 650 samples randomly selected from a nationwide Thailand population confirmed that the 6 most common haplotypes (from highest to lowest frequency) were NAT2*4 (35.46%), NAT2*5B (9.77%), NAT2*6A (33.85%), NAT2*7B (18.15%), NAT2*12A (0.62%), and NAT2*13A (1.77%). Three rare haplotypes (NAT2*5C (0.23%), *7C (0.08%), and *11A (0.08%)) were also found. The frequencies and 95% CIs of these nine *NAT2* haplotypes are shown in Table 3, demonstrating that NAT2*4 and NAT2*6B were the most common, occurring at nearly the same frequency. Interpretation of acetylator phenotypes revealed that the frequencies of acetylator types in the Thai population were 0.165 [0.136–0.193] for RAs, 0.429 [0.391–0.467] for IAs, and 0.406 [0.368–0.444] for SAs.

**Table 3:** Distribution of *NAT2* haplotypes in the Thai population (n = 650).

		*NAT2* haplotype 2											*NAT2* haplotype	
		*4	*5B	*5 C	*6 A	*7B	*7 C	*11 A	*12 A	*13 A		n	Frequency	95% CI
*NAT2* haplotype 1	*4	93	44	1	139	78	0	1	3	9		461	0.355	0.329–0.381
	*5B		8	0	43	24	0	0	0	0		127	0.098	0.082–0.115
	*5C			0	0	1	0	0	0	1		3	0.002	0.000–0.007
	*6A				88	73	1	0	2	6		440	0.338	0.313–0.365
	*7B				0	26	0	0	3	5		236	0.182	0.161–0.204
	*7C					0	0	0	0	0		1	0.001	0.000–0.004
	*11A					0	0	0	0	0		1	0.001	0.000–0.004
	*12A						0	0	0	0		8	0.006	0.003–0.012
	*13A							0	0	1		23	0.018	0.011–0.026

## Discussion

A novel method was developed for direct genotyping of *NAT2* diplotypes using haplotype-specific primers to amplify 6 common *NAT2* haplotypes (*NAT*2*4, *5*B*, *6*A*, *7*B*, *12*A*, and *13*A*) found in a non-African population, i.e, the Thai population^5^. In this method, *TIMP1* amplification was used as an internal control to safeguard against false negatives from failure of the PCR experiment due to the reaction mix, thermal cycler, or DNA sample. When this 817-bp product is absent from any HS-PCR reaction tube, that sample should be interpreted as having an indeterminate result and retested. This quality-control step ensures that homozygote diplotype calls obtained using HS-PCR are not caused by the failure to amplify another haplotype.

A validation method showed that this HS-PCR technique for *NAT2* diplotyping provided perfect concordance of acetylator phenotype interpretation (107 RAs, 279 IAs, and 264 SAs) with the results of the reference sequencing method.

The three discordant haplotyping results between the sequencing method and this HS-PCR method were NAT2*5C vs. NAT2*5B (*n* = 3), NAT2*7C vs. NAT2*7B (*n* = 1), and NAT2*11A vs. NAT2*4 (*n* = 1). Since NAT2*5C, NAT2*5B, NAT2*7C, and NAT2*7B are slow acetylator haplotypes and NAT2*11A is a fast acetylator haplotype, interpretation of the phenotypes for these five cases was not changed, as follows: two cases of the IA type (NAT2*4/*5C = NAT2*4/*5B and NAT2*5C/*13A = NAT2*5C/*13A), two cases of the SA type (NAT2*6A/*7*C* = NAT2*6A/*7*B* and NAT2*5C/*7B = NAT2*5B/*7*B)*, and one case of the FA type (NAT2*4/*11A = NAT2*4/*4).

Because this method provides 2 individual haplotypes, it is very useful for adjusting acetylator phenotypes to alternative classifications according to new findings. For example, NAT2*5B/*5B, NAT2*5B/*6A, and NAT2*5B/*7B are suggested to be slow acetylator haplotypes, whereas NAT2*6A/*6A, NAT2*6A*7B, and NAT2*7B/*7B are ultraslow acetylator haplotypes associated with DILI^24,25^.

In this study, the *NAT2* haplotypes detected by Sanger sequencing and haplotype inference in 650 samples randomly selected from a nationwide Thailand population confirmed the pattern of the 6 most commonly found haplotypes (*4,*6B, and *7A at a higher frequency and *5,*12A, and *13A at a lower frequency) as being similar to those previously reported^23^ in a northeastern Thai sample (*n* = 235 individuals). Our study found a lower frequency of *5 but a higher frequency of *13 compared with those in the study by Kulkongviriyapan et al. (*p* < 0.05). However, compared with Sabbagh et al. (*n* = 44 Thai individuals), we did not find significant differences in the frequencies of *4, *5B, *6A, *7B, and *13. The interpretation of SA phenotypes was not different between our findings and those of Kulkongviriyapan et al. [0.406 (0.368–0.444) vs. 0.362 (0.300–0.423), respectively].

As of April 2016, 108 *NAT2* haplotypes grouped into 20 clusters were recorded^2^. A member of a *NAT2-haplotype* cluster will have a cluster-signature SNP plus other SNPs. A cluster consisting of *5–*7 and *11–*14 corresponds to members with more than 1 haplotype, while the rest are rarely found. This novel HS-PCR method for *NAT2* diplotyping does not cover the NAT2*14 cluster found in African populations; therefore, it cannot be used in such populations.

Since this method was developed based on the six most common *NAT2* haplotypes, other uncommon haplotypes cannot be clearly determined and may be misclassified. The haplotypes that can potentially be amplified by each of the six specific *NAT2*-haplotype reaction tubes are shown in supplement 1. However, the misclassifications that we observed in the Thai population were mostly within the same cluster and/or shared the same acetylator phenotype, such that clinical recommendations were unchanged. Some DNA samples with rarer *NAT2* haplotypes may generate unusual band patterns or show more than two haplotypes. In these cases, the DNA sample should be checked for possible cross-contamination at the DNA extraction or PCR step. If the result remains uninterpretable, the sample should be subjected to Sanger sequencing.

## Conclusion

This method involves direct *NAT2* diplotyping, has no risk of errors caused by statistical haplotype inference, and can be implemented in a simple molecular laboratory with a lower cost and shorter turnaround time than those for other methods. This novel HS-PCR method is the first step toward enabling the routine use of *NAT2* acetylator status as an indicator in clinical practice.

### Disclaimer

A patent has been filed for the primer set designed/developed in this study in Thailand (No. 1601001130) and internationally (PCT/TH2017/000014).

## Supplementary information

*NAT2*-haplotypes potentially be amplified by each of the 6 HS-PCR tube.
