# Supplementary material for: Haplotype-specific PCR for NAT2 diplotyping
Source: Hum Genome Var. 2020 May 11;7:13. doi: 10.1038/s41439-020-0101-7 (PMC7214404; doi:10.1038/s41439-020-0101-7)
Supplement: Supplementary file 1 — NAT2-haplotypes potentially be amplified by each of the 6 HS-PCR tube. [file 41439_2020_101_MOESM1_ESM.docx]

**Supplement 1.** *NAT2*-haplotypes potentially be amplified by each of the 6 HS-PCR tube.

Reference: Boukouvala S. Human NAT2 Alleles (Haplotype). Available at: http://nat.mbg.duth.gr/Human%20NAT2%20alleles_2013.htm. [Accessed 11 October 2019]

***NAT2-haplotypes* that are positive with *NAT2-*4* HS-PCR.**

***NAT2-haplotypes* that are positive with *NAT2-*5B* HS-PCR.**

***NAT2-haplotypes* that are positive with *NAT2-*6A* HS-PCR.**

***NAT2-haplotypes* that are positive with *NAT2-*7B* HS-PCR.**

***NAT2-haplotypes* that are positive with *NAT2-*12A* HS-PCR.**

***NAT2-haplotypes* that are positive with *NAT2-*13A* HS-PCR.**
